# Aflatoxin B1 Occurrence in Children under the Age of Five’s Food Products and Aflatoxin M1 Exposure Assessment and Risk Characterization of Arab Infants through Consumption of Infant Powdered Formula: A Lebanese Experience

**DOI:** 10.3390/toxins14050290

**Published:** 2022-04-19

**Authors:** Rouaa Daou, Maha Hoteit, Khlood Bookari, Majid Al-Khalaf, Sahar Nahle, Ayoub Al-Jawaldeh, Mohamad Koubar, Samah Doumiati, André EL Khoury

**Affiliations:** 1 Centre d’Analyses et de Recherche (CAR), Unité de Recherche Technologies et Valorisation Agro-Alimentaire (UR-TVA), Faculty of Sciences, Campus of Sciences and Technologies, Mar Roukos, Saint-Joseph University of Beirut, Beirut 1104 2020, Lebanon; rouaa.daou@net.usj.edu.lb (R.D.); sahar.nahle@hotmail.com (S.N.); 2 Faculty of Public Health, Lebanese University, Beirut 6573, Lebanon; mkoubar@ul.edu.lb (M.K.); sdoumiati@ul.edu.lb (S.D.); 3 PHENOL Research Group (Public HEalth Nutrition Program-Lebanon), Faculty of Public Health, Lebanese University, Beirut 6573, Lebanon; 4 Lebanese University Nutrition Surveillance Center (LUNSC), Lebanese Food Drugs and Chemical Administrations, Lebanese University, Beirut 6573, Lebanon; 5 National Nutrition Committee, Saudi Food and Drug Authority, Riyadh 344, Saudi Arabia; kbookari@taibahu.edu.sa (K.B.); mmkhalaf@sfda.gov.sa (M.A.-K.); 6 Executive Department of Monitoring and Risk Assessment, Food Sector, Saudi Food and Drug Authority, Riyadh 3292, Saudi Arabia; 7 Department of Clinical Nutrition, Faculty of Applied Medical Sciences, Taibah University, Madinah 344, Saudi Arabia; 8 Research Laboratory of Microbiology, Department of Life and Earth Sciences, Faculty of Sciences, Lebanese University, Beirut 6573, Lebanon; 9 World Health Organization Regional Office for the Eastern Mediterranean, Cairo 11371, Egypt; aljawaldeha@who.int

**Keywords:** Aflatoxin B_1_, Aflatoxin M_1_, occurrence, exposure, infants, Lebanon

## Abstract

Aflatoxin M1 (AFM1) is a salient metabolite that can be used to assess Aflatoxin B_1_ (AFB1) exposure in humans and animals. The carcinogenic potency of AFB1 and AFM1 was severely reported. The aims of this study were (1) to survey the contamination level of AFM1 in the most traded infant powdered formula brands (IPF) (*n* = 42) along with the AFB1 level in under 5’s children food brands (biscuits, cornflakes, and cereals) (*n* = 42) and (2) to assess the estimated daily intake (EDI), the hazard quotient (HQ) and the margin of exposure (MOE) of AFM1 among infants (0–12 months) in Lebanon. All of the samples were analyzed using ELISA technique. AFB1 was below detection limit in all of the children’s food brands samples. Out of 42 IPF samples 9.5% were AFM1-positive in the range of 29.54–140.16 ng/L and exceeded the maximum tolerable limit (MTL) set by the European commission (25 ng/kg). The overall average contamination level was 5.72 ± 0.014 ng/L. The EDI of AMF1 for male was in the range of 0.37–0.78 ng/kg/b.w./day and 0.40–0.87 ng/kg/b.w./day for females. Similarly, the HQ calculation resulted in an average of 3.05 for males and 3.28 for females. MOE calculations were far lower from 10,000 in both genders which indicates a high risk of genotoxicity and carcinogenicity. Our findings show that AFM1’s EDI, HQ and MOE scored high among Lebanese infants. As infants consume more IPF relative to their body weight, the persistence of IPF with high AFM1 levels threatens their health. Thus, infant’s exposure risk to AFM1 in IPF should be a continuous focus of attention.

## 1. Introduction

Aflatoxins, produced by the action of the filamentous fungi *Aspergillus* spp. mainly *A. flavus* and *A. parasiticus*, represent a global public health and economic concern as they are responsible for significant adverse health and economic issues affecting consumer’s health. The prevalence of mycotoxin contamination of the global food crops ranged between 60% to 80% [1]. Poor practices throughout the whole food production chain starting from agricultural practices applied on field till ones applied later in stages of storage and processing could further promote fungal growth and germination leading, therefore, to the production of aflatoxins including B, M and G series [2]. However, among those, the most toxic and frequent type is aflatoxin B_1_ (AFB1). Chronic exposure to AFB1 could lead to several health effects mainly to the liver since it is a profound hepatotoxic and hepatocarcinogenic agent [3]. Upon its ingestion AFB1 is metabolized in the liver where it gets converted into aflatoxin-8, 9-epoxide that is highly reactive and that can bind to DNA or protein molecules in the liver causing toxic effects that could lead eventually to hepatocellular carcinoma [4,5]. On the other hand, AFB1 can be hydroxylated into the less toxic form of aflatoxin M_1_ (AFM1) that gets secreted by the mammary glands and contaminates milk and dairy products [6]. Therefore, due to their profound effects, aflatoxins have been classified by the International Agency for Research on Cancer (IARC) as “carcinogenic to humans” (group 1 carcinogens) [7]. Moreover, impaired child growth can also be caused by being exposed to aflatoxins as for example, a follow-up study in Benin, showed that there was a strong negative correlation between aflatoxin-albumin adducts and height increase in children over eight months period [8,9]. Additionally, evidence from West Africa showed aflatoxins chronic exposure to be directly linked with poor growth characterized by underweight and stunting in children below the age of five years, although the mechanism by which growth impairment is caused is still unknown [10,11]. Evidence from a study conducted in Gambia showed that aflatoxin exposure was inversely related to weight-for-length, weight-for-age, and length-for-age scores for children between 6 and 18 months [12]. Additionally, research findings in Nigeria reported that higher concentrations of blood aflatoxin-lysine adducts were found in children suffering from kwashiorkor [13]. Another proposed mechanism of childhood growth impairment by aflatoxins was reported in Kenyan children; this study estimated that 16% of the cases of reduced height could be attributable to the reduction in insulin-like growth factor hormone (IGF) [14]. However, additional studies are still needed in this domain to fully understand the mechanisms that lead to impaired child growth associated with aflatoxins exposure and implement interventions accordingly.

Infants (0–12 months) are the foremost milk consumers and the most vulnerable group in the population, which make them more susceptible to the adverse effects of AFM1 (particularly hepatocellular carcinoma) [15]. Therefore, the incidence of AFM1 in milk and milk products is a serious health concern. The levels of AFM1 in milk depend on the initial levels of AFB1 in feed [16]. Moreover, AFM1 is heat stable and can resist various thermal treatments, and is almost entirely retained in pasteurized, and infant powdered formula (IPF) [17]. Due to its relation to environmental factors, preventing the incidence of AFB1 contamination in feed cannot be easily attained, accordingly, zero AFM1 contamination levels cannot be practically achieved at all times. However, exposure to it can be controlled by restricting the level of AFB1 in feeds through imposing strict regulations on AFB1 in feed and on AFM1 in milk and milk products. Accordingly, the European Union has established the strictest maximum levels for AFM1 (0.05 µg kg^−1^) in raw milk, heat-treated milk and milk for the manufacture of milk-based products and 0.025 µg kg^−1^ in infant formula, including starter milk and follow-up milk [18] with a tolerable daily intake (TDI) of 0.2 ng/kg b.w./day [19]. Similarly, Lebanon has set standards for aflatoxins where the maximum tolerable limit (MTL) for AFM1 in raw milk, heat-treated milk, and milk for the manufacture of milk-based products is 0.05 µg kg^−1^ [20]. 

Recent studies in the Arab countries reported the increasing incidence AFM1 in IPFs, with many samples exceeding the international MTL [21,22,23,24]. These reports drew the attention to the increased health and life-threatening risks that infants might be exposed to upon consuming such contaminated products. Based on an extensive search, and to our knowledge, no national data investigated the occurrence of AFB1 in children under the age of five’s food brands. Moreover, only a single study discussed the contamination of IPF brands with AFM1 in Lebanon [25] For this reason, this study aims to (1) survey the contamination level of AFM1 in the most traded IPF (*n* = 42) along with the AFB1 level in children under the age of five’s food brands (biscuits, cornflakes, and cereals) (*n* = 42) and (2) to assess the estimated daily intake (EDI), the hazard quotient (HQ) and the margin of exposure (MOE) of AFM1 through the consumption of IPF among infants (0–12 months) in Lebanon.

## 2. Results

### 2.1. Occurrence of AFM1 and AFB1 in IPF and Children’s Food Products

The occurrence of AFM1 in IPF and that of AFB1 in children under the age of five’s food products are presented in Table 1. Amongst the 42 collected samples of children under the age of five’s food products (biscuits, corn flakes and cereals products), AFB1 was shown to be below detection limit in all samples. On the other hand, the mean concentration of AFM1 in starter formula (3.5 ± 0.06 ng/L) was lower than that reported in follow-up formula (7.7 ± 0.07 ng/L). Overall, the mean contamination in 42 samples of IPF was 5.72 ± 0.014 ng/L, far below the maximum tolerable limit (MTL) set by the European commission (25 ng/kg). It was shown that 9.5% of these samples exceeded the MTL (Table 1). 

### 2.2. AFM1 Dietary Exposure Assessment

AFM1′s risk assessment through IPF dietary exposure estimated the magnitude and the probability of the harmful effect from AFM1 on infants (0–12 months). Exposure assessment, as one component of risk assessment methodology, combines AFM1 levels in IPF with consumption patterns, and therefore, provides valuable information for risk management in later stages. Exposure to AFM1 from IPF are shown in Table 2. The consumption patterns were derived from the guidelines on the amount and number of feedings per day for every infant age group detailed on the infant formula packages labels. The EDI reported in this study was on average reported to be 0.61 and 0.66 in males and females, respectively. The HQ values at all ages and in both males and females were shown to be over 1. MOE values were shown to be below 10,000 among both genders (Table 2). Infants aged 0–6 months had the highest risk of exposure to AFM1 in IPF, with an EDI of 0.67 and 0.71 ng/kg/b.w./day for males and females, respectively. Similarly, the AFM1′s HQ of males and females among infants aged 0–6 months were higher than those aged 6–12 months (3.37 ng/kg/b.w./day and 3.57 ng/kg/b.w./day versus (vs.) 2.67 ng/kg/b.w./day and 2.93 ng/kg/b.w./day, respectively). It is noteworthy that both sub-categories of infants had a mean MOE that was far below 10,000 in both genders (187.03 in males and 173.77 in females).

### 2.3. Calculation of Extrapolated Values of Aflatoxin B1 Concentration in Feeds 

According to Equation 4, the range of feed extrapolated AFB1 content of starter milk formula ranged between 1.91–2.5 µg AFB1/kg. On the other hand, the range of feed extrapolated AFB1 content of follow-up milk formula and all of the IPF samples was 1.84–8.76 µg AFB1/kg, of which the upper value exceeded the maximum allowed level which is 5 µg AFB1/kg feed for dairy animals [15].

## 3. Discussion

The current study showed that AFB1 in all of the children’s food brands samples were below the limit of detection. As for AFM1, out of 42 IPF samples, 9.5% were found to contain AFM1 in the range of 29.54–140.16 ng/L and exceed the MTL set by the European Commission at 25 ng/kg. The overall mean AFM1 contamination level was found in this study to be 5.72 ± 0.014 ng/L. This study also reported the exposure to AFM1 from infant formula and assessed the risk resulting from this exposure. Accordingly, the EDI of AFM1 was in the range of 0.37–0.78 ng/kg b.w./day and 0.40–0.87 ng/kg b.w./day for males and females, respectively. Similarly, the HQ calculation resulted in an average of 3.05 and 3.28 for males and females. MOE calculations were reported to be much lower than 10,000 for both genders which indicates a high risk of genotoxicity and carcinogenicity. 

Due to the limited AFB1 concentration data for children under the age of five’s food products, these food categories were not taken into account for risk assessment. The presence of AFM1 in 9.5% of IPF samples in the current study is most probably the consequence of feeding dairy cows a diet contaminated with AFB1. Overpassing the maximum allowed level which is 5 µg AFB1/kg feed for dairy animals, the feed extrapolated AFB1 content of milk of the IPF samples sold in Lebanese pharmacies was 1.84–8.76 µg AFB1/kg. Due to the thermostability of AFM1 that is characterized by resistance to chemical and physical treatment, monitoring AFB1 levels in animal feeds is essential to minimize AFM1 contamination in IPF [26]. The overall mean AFM1 contamination of the 42 IPF samples reported in this study was 5.72 ng/L that is lower than the European and Lebanese MTL [18,20]. 

The mean concentration of AFM1 in the present study (5.72 ng/L) is lower than the previously reported prevalence in 2019 in Lebanon by Elaridi et al., 2019 (20.1 ng/L) [25]. Moreover Elaridi et al. (Lebanon) reported higher contamination levels with AFM1 that was detected in 88% (*n* = 42 samples) with 31% of the samples having concentrations that exceeded the MTL of 25 ng/L [25]. Aflatoxin contamination of Lebanese foods and products was severely reported in many national studies. Recently, our research group published the first national database that encompasses full details about the contamination and exposure of all edible products in Lebanon. This database shows that aflatoxin contamination is a national public health and economic concern [27].

In the Arab countries, the quantity of research in regional aflatoxin contamination of food has substantially increased over the past two decades [28].According to a recent systematic review and meta-analysis, the prevalence of aflatoxin M_1_ (AFM_1_) of the consumed cow milk in the Arab region ranged between 11% to 85% with the highest in Palestine [28]. Moreover, the prevalence of aflatoxin M_1_ in the raw milk of Lebanon, Palestine, Egypt, and Syria was identified as 67%, 85%, 38%, and 14%; pasteurized milk, in Lebanon, was 36%, and finally UHT milk in Saudi Arabia was 62%, respectively. As for the IPF, studies investigating the prevalence of AFM1 contamination are scarce. 

### 3.1. Comparison with International Studies

In comparison to international studies, our results were lower than Argentina in which the number of samples exceeding the MTL was 100% and the mean level of contamination was 393 ng/L [29]. Similarly, the number of samples exceeding the MTL in 2 studies in Brazil (100%, mean = 346 ng/L) [29]; 44%; mean = 24 ng/L [30], in India (100%) [31]; in Mexico (20%; mean = 40 ng/l) [32]; in Pakistan (30.8%, mean = 20 ng/L) [33], in Turkey (45%, mean = 60 ng/L) [34] were higher than our findings (9.5%). 

On the other hand, the number of samples that exceeded the MTL in our study surpassed the findings reported in Brazil (5.2%, mean = 26 ng/L) [35], Iran (0%, mean = 7.31 ng/L) [36] and (0%, mean = 21.7 ng/L) [37], Italy (4.3%, mean = 32.2 ng/L) [17]; 0%, mean = 0 ng/L), Portugual (0%, mean = 12.1%) [38], Serbia (0%, mean = 0.9 ng/L) [39], Spain (0%, mean = 3.1 ng/L) [40], Turkey (0%, mean = 18 ng/L [24]; 0%, mean = 8.9 ng/L [41]; 0%, mean = 0 ng/L [42]. 

### 3.2. Comparison with Other Arab Countries

In comparison with Arab countries, Abbas et al. (2001) reported no contamination for IPF in [43] Kuwait [44]. The number of samples which exceeded the MTL in Jordan (85%, mean = 120.6 [45]; 48.3%, mean = 74.2 [46] was higher than that reported in our study. Furthermore, the results reported in Qatar were in concordance with our findings (9.5%) of which 8–11% of the IPF samples were contaminated with AFM1 [47]. In Egypt, the mean concentrations of AFM1 in IPF was 9.796 ± 1.036 ng/L and the prevalence of contamination was close to 43% [21]. However, there was scarce data concerning the number of samples that exceeded the MTL in these studies (Table 3). 

### 3.3. Exposure Assessment of Lebanese Infants to AMF1 from IPF

To our knowledge, the current study is the first ever study to assess the EDI, HQ and MOE of AFM1 by Lebanese infants. Our findings indicated a high EDI (>0.2 ng/kg b.w./day) and high HQ (>1) for both age groups (0–6 months and 6–12 months). On the other hand, it was observed that MOE levels were far below 10,000 indicating serious health risk for infants in Lebanon. In comparison to international studies, our results were approximately similar to a French study that showed that the maximum EDI was 0.48 and 0.55 ng AFM1/kg.b.w./day for infants in Catalonia and France [48]. Moreover, a Brazilian study showed higher EDI than our results, where EDI value was 3.7 ng AFM1/kg b.w./day [49]. Many other studies reported several EDI data in Spain, Argentina, and Thailand with values of 0.16–3.70 ng AFM1/kg b.w./day [50,51,52]. On the other hand, our findings were higher than the EDI values (0.078–0.306 ng AFM1/kg) reported in Brazil [30]. At the Arab countries level, the average daily exposure of AFM1 through the consumption of IPF for Egyptian infants was higher than that reported in the current study (8.170 ng) [21]. Moreover, the EDI findings in Jordan were 1.573 and 1.551 ng/kg b.w./day for 6- and 12-months infants age, respectively; values that surpassed our findings [46].

The HQ in this study was above 1, which also indicates non-tolerable exposure levels through infant formula in Lebanon.

As for risk characterization, the risk of exposure to AFM1 through IPF consumption was characterized using MOE. MOE calculations in the current study were far lower from 10,000 in both genders which indicates a high risk of genotoxicity and carcinogenicity. Very few studies reported MOE to AFM1 among their population. Nevertheless, our findings were higher than the values reported in Serbia of which the MOE to AFM1 was far lower from 10,000 among children [53]. 

### 3.4. Calculation of Extrapolated Concentration of Aflatoxin B1 in Animal Feed

Due to its heat-stability, AFM1 cannot be removed or eliminated by chemical or physical treatment methods, thus it is recommended to monitor AFB1 levels in animal feeds as an effective measure to control contamination of AFM1 in milk and thus IPF [26]. AFB1 is allowed at a maximum level of 5 µg AFB1/kg feed for dairy animals such as cattle [15]. Our findings showed that the range of feed extrapolated AFB1 content of IPF was 1.84–8.76 µg AFB1/kg, of which the upper value exceeded the maximum allowed level [15]. In Lebanon, no studies reported such type of extrapolation. In comparison to our results, high-extrapolated AFB1 values for pasteurized cow’s milk only indicated a range from 0.6 to 8.1 and 0.9 to 13.5 µg AFB1/kg, respectively [54]. The data reported in our study highlights that the best measure to control AFM1 in IPF should start at the farm level to ensure the safety of milk used in IPF processing through controlling the contamination level of AFB1 in feeds by applying good agricultural, storage, and hygiene practices at the farm level. 

As for AFB1, it was not detected in any of the samples tested in this study. In Lebanon, no previous studies addressed infant food products specifically. However, AFM1 was tested in breakfast cereals in two studies of which AFB1 was not detected in any sample in the first one while it was detected in 100% of samples in the second study with a mean of 0.158 µg/kg [55,56]. Limited number of studies investigating the occurrence of AFB1 in infant food products are summarized in Table 4.

In comparison to international studies, our results were lower than Spain and Iran in which the percentage of samples that exceeded the MTL was reported to be 10% and 52% with mean contamination levels of 30 and 0.2602 µg/kg, respectively [57,58]. Additionally, higher mean contamination levels of AFB1 were found in Spain and Turkey at 0.09 and 0.8 µg/kg, respectively [34,59]. Similar to our findings, the samples collected from Czech Republic and Indonesia had AFB1 at undetectable levels [60,61].

Lebanon, a middle-income country, is rapidly sinking into poverty as it faces a triple shock from the unprecedented economic crisis, the impact of COVID-19 on employment and public health, and the consequences of Beirut port explosions. AFM1′s EDI and HQ scored high among Lebanese infants therefore presenting health risks. The findings in our report highlight the importance of controlling the importation of IPF from countries controlling AFM1 and AFB1 levels in children’s milk and food to avoid flooding Lebanese pharmacies and markets with AFM1 and AFB1 contaminated food products. If this issue was left untreated, infants (0–12 months) group in Lebanon as well in all other Arab countries can be at higher risk of mycotoxin-attributable health risks.

## 4. Strength and Limitations

This is the first study to assess the risk assessment and risk characterization to AFM1 in infants’ population through IPF consumption in Lebanon. Moreover, this is the first study assessing the AFB1 concentration in children’s food products. The comparison of our findings using harmonized occurrence and exposure data collected globally, ensure a uniform and comprehensive report. The limitations of this study were as follows. First, the sample size is relatively small. However, those samples represent the majority of the brands available on the Lebanese market. Second, the data on the weight of infants used for exposure calculation was not based on Lebanese studies due to their unavailability. Third, the consumption patterns were derived from information on the infant formula label instead of actual consumption due to the lack of research in this area in the Lebanese context. Finally, the total exposure to AFM1 was not assessed through the dietary intake of other food groups that may be contaminated with AFM1 as well. Therefore, future studies should concentrate on generating data regarding Lebanese infants’ growth patterns and milk consumption, and should accordingly use this data to assess AFM1 exposure from infant formula, breast milk, and other various food products.

## 5. Conclusions

Since aflatoxins presence in food was reported to cause several adverse health and economic effects, it is particularly important to control their contamination in the food chain through enforcing regulations of AFB1 and AFM1 in feed and food, respectively. In this study, AFM1′s EDI, and HQ scored high among Lebanese infants so the risk from exposure prevails. This risk is particularly due to the fact that chronic exposure in such an age group is considered hazardous especially since they are more susceptible to higher health effects due to their lower body weights and immune status. Therefore, infant’s exposure risk to AFM1 in IPF at any level is considered unacceptable and poses health risks. Hence, AFM1 in IPF should be a continuous focus of attention in Lebanon and other Arab countries as well that usually import the same formula brands.

## 6. Materials and Methods

### 6.1. Sampling Methods

Pre-packaged IPF (starter and follow up types) (*n* = 42) marketed frequently in the Arab countries and food products frequently consumed by children (biscuits, cornflakes, and cereals) (*n* = 42) were randomly collected from Lebanese pharmacies and supermarkets, respectively. Samples were checked to ensure that they didn’t exceed their expiry dates, additionally, samples were stored in controlled humidity and temperature conditions until the time of analysis. Each sample was tested in triplicates on the same ELISA plate. To ensure quality of testing the same pipette was used for each sample. The means and standard deviations (SD) were calculated for each food product.

### 6.2. Enzyme Linked Immunosorbent Assay (ELISA) Quantification

#### 6.2.1. AFM1

RIDASCREEN^®^ Aflatoxin M1 kit (R1121) was purchased from R-biopharm (Darmstadt, Germany). First, 10 g of powdered milk were mixed with 100 mL of distilled water. Then for the ELISA test, the instructions on the manual were followed in which the wells were incubated in the following order: antibodies, samples and standards, conjugate, and substrate/chromogen. After each step of incubating with antibodies, samples and standards, and conjugate, washing with Phosphate Buffered Saline (PBS) was done, so in total 3 washings were applied and each washing was repeated two times. Finally, a stop solution was added that turned the color of the substrate/chromogen from blue to yellow which was measured using spectrophotometry at 450 nm. A standard curve was constructed and then the concentration of AFM1 was quantified in each sample. The limit of detection as supplied by the manufacturer was 0.005 µg/L.

#### 6.2.2. AFB1

RIDASCREEN^®^ Aflatoxin B1 30/15 kit (R1211) was purchased from R-biopharm (Darmstadt, Germany). Infant food products brands were purchased as three packages from the same brand with different lot numbers, the products were mixed together, ground, and homogenized by thorough mixing. After that, 5 g were withdrawn and were mixed with 25 mL of 70% methanol, shaken vigorously for 3 min, and centrifuged at 3500× *g* for 10 min. Afterwards 1 mL of the supernatant was extracted and diluted with 1 mL of distilled water. Then for the ELISA test, the instructions on the manual were followed, first, 50 µL of the standards and samples were added to the wells, followed by conjugate and antibodies. Then after incubation and PBS washing steps, a substrate/chromogen was added to each well and incubated. Finally, a stop solution was added that changed the color in the wells from blue to yellow which was measured using spectrophotometry at 450 nm. A standard curve was constructed and then the concentration of AFB1 was quantified in each sample. This was repeated three times. The limit of detection as supplied by the manufacturer was 0.001 µg/L.

### 6.3. Exposure Assessment 

Exposure was calculated for 42 IPF (starter and follow-up types) and for infants (0–12 months) according to their gender, age and normal body weight as expressed by Centers for Disease Control and Prevention (CDC) to highlight the differences in exposure [62]. The EDI of AFM1 (expressed as ng kg/b.w./day) was calculated based on the concentration of AFM1 detected and the intake rate of analyzed IPF, according to Equation (1): [63]

Equation (1):

EDI (ng/kg b.w./day) = MC_AFM1_ (ng/L) × IPFI (L/day) BW (kg)(1)

where EDI is the estimated daily intake of AFM1, MC_AFM1_ is the mean concentration of AFM1 in IPF reported in this study, IPFI is the mean daily infant powdered formula intake that is shown in Table 2 and was based on the IPF’s label instructions. The AFM1 Hazard Quotient (HQ), calculated as the ratio between average exposure and the toxicological threshold, was reported. A ratio of HQ > 1 implies a non-tolerable exposure level [64].To calculate AFM1 HQ the following equation reported by Sharafi et al. was used as well [63]. 

Equation (2):

Hazard Quotient (HQ) = EDI (ng/kg b.w./day) RFD (ng/kg b.w./day)(2)

where RFD in this equation was reported to be 0.2 ng/kg b.w./day by Sharafi et al. and it was calculated according to a threshold value that is equivalent to the amount of AFM1 that induces tumor in half of laboratory animals and that was divided by an uncertainty factor of 50,000.

Furthermore, the risk characterization originating from the oral exposure to aflatoxins was calculated using a qualitative margin of exposure (MOE) approach established by EFSA [64]. Thus, a third equation also reported by Sharafi et al. was used to calculate the MOE [63].

Equation (3):

Margin of Exposure (MOE) = 570 ng/kg b.w./day EDI (ng/kg b.w./day) (3)

The value of 570 ng/kg b.w./day in this equation is equal to the benchmark dose lower confidence limit (BMDL10) for 10% increased cancer risk in rats in a two-year research study. Accordingly, a calculated MOE value lower than 10,000 implies that exposure to a carcinogenic and genotoxic substance contributes to the risk of hepatocellular carcinoma and is of concern to public health. 

#### Calculation of Extrapolated Values of Aflatoxin B1 Concentration in Feeds 

A linear relationship was proposed between the concentration of AFB1 in feeds consumed by animals such as cows and AFM1 in milk and it has been reported that only 1.6% of ingested AFB1 is bio transformed to AFM1 by the dairy cattle [65]. According to Price et al. (1985) the expected concentrations of AFB1 in animal feeds can be back calculated from the concentrations of AFM1 in IPF samples [66].Therefore, the values of AFB1 contamination in dairy animal feeds were back calculated by the formula given below:

Equation (4):

AFB1 (μg/kg) = [AFM1 (ng/kg) × 100]/1.6/1000.(4)

## Figures and Tables

**Table 1:** Occurrence of AFM1 in infant powdered formula and AFB1 in children under the age of five’s food products.

Type	*n*	Positive Samples *n* (%)	>MTL ^a^ *n* (%)	Mean ± SD ^b^	Range of Contamination
AFM1					
Infant powdered formula	42	4 (9.5%)	4 (9.5%)	5.72 ± 0.014 ng/L	29.54–140.16 ng/L
Starter formula (0–6 months)	20	2 (10%)	2 (10%)	3.5 ± 0.06 ng/L	30.7–40.15 ng/L
Follow up formula (6–12 months)	22	2 (9%)	2 (9%)	7.7 ± 0.07 ng/L	29.54–140.16 ng/L
AFB1					
Children under the age of five’s food products	42	0	0	0 µg/kg	BDL

^a^ MTL for AFM1 in infant formula is 25 ng/kg and for AFB1 in cereal-based food intended for consumption by infants is 100 ng/kg. ^b^ The mean reported in this table represents contamination in all samples not only positive ones, *n*: number of samples, BDL: below detection of limits

**Table 2:** Exposure and risk characterization to AFM1 among males and females infants (0–12 months) through IPF intake at different ages calculated through EDI, HQ and MOE, respectively.

Age	IPF Consumption	Average Weight * (kg)		EDI (ng/kg b.w./Day)		HQ		MOE	
	Ml/Day	Male	Female	Male	Female	Male	Female	Male	Female
0–1 weeks	420	3.8	3.7	0.39	0.40	1.93	1.99	1473.47	1434.69
1–4 weeks	540	4.3	4.5	0.44	0.42	2.20	2.10	1296.83	1357.14
2–8 weeks	720	5.3	5.2	0.48	0.48	2.38	2.42	1198.81	1176.19
2–3 months	750	6.5	5.9	0.40	0.44	2.02	2.22	1411.43	1281.14
3–5 months	900	7.5	6.9	0.42	0.46	2.10	2.28	1357.14	1248.57
5–6 months	900	8.5	7.7	0.37	0.41	1.85	2.05	1538.10	1393.33
6–8 months	945	9.3	8.4	0.78	0.87	3.91	4.33	728.51	658.01
8–10 months	945	10.2	9.3	0.71	0.78	3.57	3.91	799.01	728.51
10–12 months	945	10.9	10	0.67	0.73	3.34	3.64	853.84	783.34
**Age Categories**									
0–6 months	705	5.98	5.6	0.67	0.71	3.37	3.57	169.15	159.72
6–12 months	945	10.13	9.23	0.53	0.59	2.67	2.93	213.71	194.73
Average	785	7.4	6.84	0.61	0.66	3.05	3.28	187.03	173.77 *

* https://www.cdc.gov/growthcharts/html_charts/wtageinf.htm (accesed on 12 March 2022). It was based on 75 percentile.

**Table 3:** AFM1 contamination in infant formula samples from different countries around the world.

Country	Year	Number of Samples	Positive Samples	>MTL	Mean± SD (ng/L)	Range (ng/L)	Method	Reference
Argentina	2013	1	100%	100%	320	N.D.	HPLC	[29]
Brazil	2013	9	100%	100%	346	N.D.	HPLC	[29]
	2016	16	44%	N.D.	24 ± 10	N.D.–46	HPLC	[30]
	2018	38	32%	5.2%	26 ± 19	13–67	ELISA	[35]
Egypt	2011	125	43%	N.D.	9.8 ± 1	5–25	ELISA	[21]
India	2013	18	100%	100%	N.D.	501–713	ELISA	[31]
Iran	2007	120	96.6%	0	7.31 ± 3.9	1–14	ELISA	[36]
	2020	29	3.4%	0	21.7	N.D.	HPLC	[37]
Italy	2001	92	53%	4.3%	32.2 ± 3.68	<1–23.5	HPLC	[17]
	2009	185	1%	N.D.	14	11.8–15.3	HPLC	[54]
	2014	13	0	0	0	0	LC-MS/MS	[43]
Jordan	2016	20	100%	85%	120.26 ± 33.54	16.55–154.14	ELISA	[45]
	2019	120	48.3%	48.3%	74.2 ± 7.54	5–213.84	ELISA	[46]
Kuwait	2001	17	0	0	0	0	HPLC	[44]
Lebanon	2019	42	88%	31%	20.1 ± 1.3	0–48.1	ELISA	[25]
Mexico	2019	55	20%	20%	40 ± 99	40–450	HPLC	[32]
Pakistan	2017	13	53.8%	30.8%	20	6–108	ELISA	[33]
Portugal	2010	7	85.7%	0	12.1	7–41	HPLC	[38]
Qatar	2018	12	33%	N.D.	N.D.	N.D.	HPLC	[47]
Serbia	2015	21	4.7%	0	0.9	20	HPLC	[39]
	2021	92	15.2%		10 ± 0.002	8–14		[53]
Spain	2010	69	37.6%	0	3.1 ± 0.6	0.6–11.6	HPLC	[40]
Turkey	2007	29	45%	45%	60 ± 30	N.D.	ELISA	[34]
	2012	62	16.7%	0	18	16–22	HPLC	[24]
	2013	84	38.1%	0	8.9 ± 6	5.5–20.1	ELISA	[41]
	2014	33	0	0	0	0	ELISA	[42]

N.D.: no data available in the study.

**Table 4:** AFB1 contamination in infant and children food samples from different countries around the world.

Country	Year	Number of Samples	Positive Samples	>MTL	Mean ± SD (μg/kg)	Range (μg/kg)	Method	Reference
Czech Republic	2010	34	0	0	0	0	LC-MS/MS	[60]
Indonesia	2004	12	0	0	0	0	ELISA	[61]
Iran	2017	48	68.7%	52%	2.60 ± 4.06	0.025–15.15	HPLC	[58]
Lebanon	2000	30 *	0	0	0	0	N.D.	[55]
	2021	10 *	100%	0	0.158	0.14–0.17	HPLC	[56]
Portugal	2010	20	30%	0	N.D.	0	HPLC	[38]
Spain	2010	91	46%	N.D.	0.09 ± 0.4	N.D.	HPLC	[59]
	2019	60	20%	10%	0.03 ± 0.05	0.06–0.23	HPLC	[57]
Turkey	2007	25	88%	N.D.	0.8 ± 0.44	N.D.	ELISA	[34]

N.D.: No data available. * Cornflakes samples.

## Data Availability

The data presented in this study are available within these article.
